# Biocomposite Polyvinyl Alcohol/Ferritin Hydrogels with Enhanced Stretchability and Conductivity for Flexible Strain Sensors

**DOI:** 10.3390/gels11010059

**Published:** 2025-01-11

**Authors:** Qiang Fu, Junxiao Tang, Weimin Wang, Rongjie Wang

**Affiliations:** 1 China Shipbuilding Industry Corporation, Research Institute 712, Wuhan 430064, China; 13429877918@139.com; 2 State Key Laboratory of Advanced Technology for Materials Synthesis and Processing, Wuhan University of Technology, Wuhan 430070, China; tangjunxiao@whut.edu.cn (J.T.); wangwm@hotmail.com (W.W.); 3 Hubei Longzhong Laboratory, Xiangyang 441022, China

**Keywords:** polyvinyl alcohol, ferritin, biocomposite hydrogel, flexible, strain sensor

## Abstract

Protein-based hydrogels with stretchability and conductivity have potential applications in wearable electronic devices. However, the development of protein-based biocomposite hydrogels is still limited. In this work, we used natural ferritin to develop a PVA/ferritin biocomposite hydrogel by a repetitive freeze–thaw method. In this biocomposite hydrogel, ferritin, as a nano spring, forms a hydrogen bond with the PVA networks, which reduces the crystallinity of PVA and significantly improves the stretchability of the hydrogel. The fracture strain of the PVA/ferritin hydrogel is 203%, and the fracture stress is 112.2 kPa. The fracture toughness of the PVA/ferritin hydrogel is significantly enhanced to 147.03 kJ/m^3^, more than 3 times that of the PVA hydrogel (39.17 kJ/m^3^). In addition, the free residues and iron ions of ferritin endow the biocomposite hydrogel with enhanced ionic conductivity (0.15 S/m). The strain sensor constructed from this hydrogel shows good sensitivity (gauge factor = 1.7 at 150% strain), accurate real-time resistance response, and good long cyclic working stability when used for joint motion monitoring. The results indicate that a PVA/ferritin biocomposite hydrogel prepared by a facile method has enhanced stretchability and conductivity for flexible strain sensors. This work develops a new method for the preparation of protein-based hydrogels for wearable electronic devices.

## 1. Introduction

As a hydrophilic three-dimensional (3D) network structure material, hydrogel has attracted more and more attention [1,2,3]. The flexible networks in the hydrogel have the ability to deform under stress, and the ions can be transported in the 3D network structure [4,5,6]. Thus, hydrogels with extensibility and conductivity are widely applied in the field of flexible electronic materials [7,8]. The strain sensors assembled by conductive hydrogels can be used to monitor the movement of human joints, such as fingers, wrists, and knees [9,10]. At present, multiple preparation methods of functional hydrogel materials were developed, such as chemical crosslinking, physical entanglement, and ionic interactions [11,12,13]. However, a hydrogel composed of a single flexible chain often has difficulty meeting the requirements of good stretchability and conductivity for strain sensors. Therefore, multiple components are designed to synergistically enhance hydrogel materials [14,15]. For example, a polyvinyl alcohol/graphene oxide hydrogel with enhanced stretchability and conductivity was prepared by combining freezing and salting out [16]. It is of great significance to choose the appropriate composition design for developing hydrogels in the application of flexible electronic devices.

Polyvinyl alcohol (PVA) hydrogel is a class of flexible material, which can be used as a strain sensor to detect mechanical deformations such as stretching, bending, or compressing [17]. These sensors utilize the unique properties of PVA hydrogels, which are water-swollen networks that exhibit excellent elasticity, biocompatibility, and environmental stability [18]. When integrated with conductive materials like carbon nanotubes or graphene, PVA hydrogels become capable of detecting minute changes in strain, offering applications in wearable electronics, soft robotics, and health monitoring systems [16,19]. For example, PVA chains are connected with graphene through tannic acid to obtain hydrogel with improved conductivity and stretchability for monitoring the movement of various parts of the human body in real-time [20]. The strain-induced changes in electrical resistance or capacitance make PVA hydrogel sensors ideal for real-time monitoring of body movements, pressure variations, or structural integrity in soft and flexible systems [21]. The advantages of PVA hydrogels include biocompatibility, high tensile strength, and the ability to operate stably in the environment, making them an attractive choice for advanced sensing applications [22]. Therefore, the development of PVA-based hydrogel materials with excellent sensing performance is of great significance in the field of flexible electronics.

In recent years, it was found that some natural proteins can be used to prepare flexible biocomposite hydrogels [23,24,25]. The hydrogen bond interactions between the functional groups of proteins and polymer chains can improve the mechanical properties of hydrogels [26,27]. For example, milk casein was introduced into polyacrylamide (PAAm) hydrogel, showing multiple adhesive properties and tough mechanical properties [28]. Silk fibroin was used to construct a biocomposite hydrogel with enhanced tensile strength for flexible strain sensors [29]. In addition, ferritin, as a natural iron storage protein, has a unique structure consisting of an inorganic iron core and a protein shell [30,31]. Among them, the protein shell of ferritin is composed of 24 α-helix structural subunits. Previous studies have found that the α-helix structure of protein shell can withstand forces like a spring [32]. The hydrogel prepared with ferritin shows enhanced toughness [33]. For example, a chemically crosslinked PAAm/ferritin hybrid hydrogel has high tensile properties and good biocompatibility for wearable strain sensors [34]. However, chemical crosslinking often produces residual by-products, such as unreacted initiators or monomers. Tough nanofiber hydrogels were prepared by incorporating ferritin and electrospinning [35]. However, this processing requires high energy consumption, and the application of hydrogels is still limited. Therefore, it is still a challenge to develop a facile method of ferritin-based biocomposite hydrogels for flexible electronic devices.

Herein, we used natural ferritin to prepare a PVA/ferritin biocomposite hydrogel by a repetitive freeze–thaw method. The functional biocomposite hydrogels were synthesized by such facile methods. The effect of ferritin on the structure and mechanical properties of hydrogels was studied. The results indicate that ferritin is uniformly dispersed in the 3D network composed of PVA chains. Ferritin can form hydrogen bonds with PVA chains and reduce the crystallinity of PVA chains. In addition, the stretchability of hydrogel is enhanced due to ferritin acting as a spring to bear the force in the hydrogel. The tensile test results indicate that after adding 5 mg/mL of ferritin, the fracture strain of the PVA/ferritin hydrogel increases from 90% to 203%, and the fracture stress increases from 59.5 kPa to 112.2 kPa. The fracture toughness of the PVA/ferritin hydrogel is significantly increased to 147.03 kJ/m^3^, more than 3 times that of the PVA hydrogel (39.17 kJ/m^3^). In addition, the biocomposite hydrogels have increased conductivity (0.15 S/m). The application performance of hydrogel in strain sensors was studied. The results show that the hydrogel has a good sensitivity (gauge factor = 1.7 at 150% strain), an accurate strain–resistance response for human joint movement monitoring, and good stability after a long cyclic working. This work could provide a facile method for a protein-based hydrogel and is expected to be used in flexible electronic devices.

## 2. Results and Discussion

### 2.1. Illustration of PVA/Ferritin Hydrogel Formation

**Figure 1:**
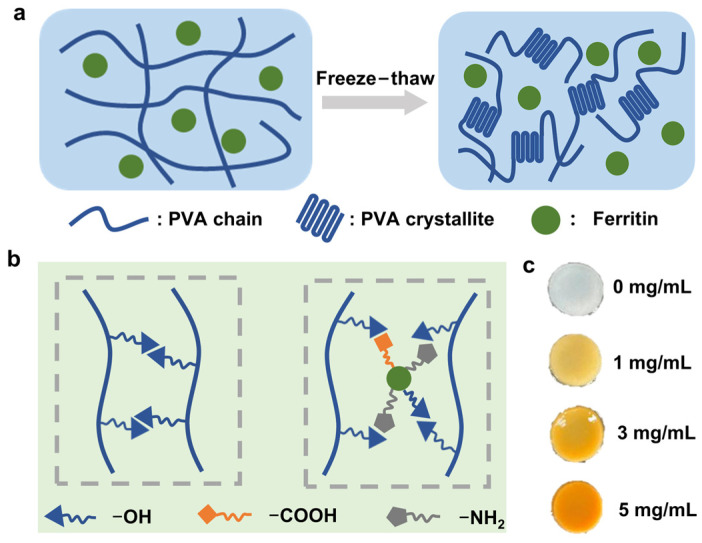
Schematic diagram and photos of hydrogels. (**a**) Schematic diagram of preparation of PVA/ferritin biocomposite hydrogels through freeze–thaw method. (**b**) Schematic diagram of hydrogen bonds in hydrogels. (**c**) Photos of hydrogel with different concentrations of ferritin.

PVA is rich in hydrophilic functional hydroxyl groups and has good water retention properties [36]. The hydrogel composed of PVA exhibits good flexibility and biocompatibility for wearable sensor applications [37,38]. Usually, the mechanical properties of PVA can be effectively improved by compounding it with organic matter [39,40]. As a natural iron storage protein, ferritin has a protein shell and an inorganic iron core [30]. The α-helix structure of the protein shell enables ferritin to dissipate mechanical forces like a spring, which can improve the tensile strength of the hydrogel [32,35]. In this work, we further developed a PVA/ferritin biocomposite hydrogel by a repetitive freeze–thaw method and explored its potential applications in strain sensors. As shown in Figure 1a, the PVA chain is used as the three-dimensional network structure of the hydrogel, and the ferritin is dispersed in the PVA chain as the nano spring to construct the PVA/ferritin biocomposite hydrogel material. The PVA is crystallized after repeated freezing and thawing, leading to the gelation of a PVA/Ferritin solution. Finally, PVA/ferritin biocomposite hydrogels were prepared through this facile process. In the PVA/ferritin biocomposite hydrogels, ferritin is uniformly dispersed in hydrogels and wrapped by a PVA chain. Figure 1b shows the chemical structure of the PVA/ferritin biocomposite hydrogel in a schematic diagram. A large number of hydroxyl functional groups of PVA chains interact through strong hydrogen bonds [41]. After introducing ferritin into PVA chains, the functional groups on the protein shell of ferritin, such as hydroxyl, carboxyl, and amino groups, are bonded with the hydroxyl groups of PVA chains through hydrogen bonding interactions. Hydrogen bonding can effectively improve the mechanical properties of hydrogels [42,43]. In addition, ferritin, as a nano spring model, is evenly dispersed in the PVA chains, which can further improve the tensile properties of PVA hydrogels. Therefore, the hydrogen bond between ferritin and a PVA chain, and ferritin as nano spring model are conducive to improving the mechanical properties of hydrogels, including tensile properties. As shown in Figure 1c, hydrogels with different concentrations of 0, 1, 3, and 5 mg/mL ferritin were prepared, respectively. With the increase in ferritin concentration, the color of the hydrogel becomes heavier.

### 2.2. FTIR and XRD Analysis of PVA/Ferritin Hydrogel

**Figure 2:**
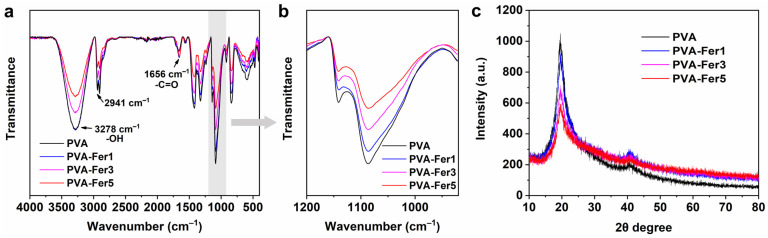
FTIR and XRD analysis of hydrogels. (**a**) FTIR spectra of hydrogels. (**b**) Local magnification FTIR spectra of hydrogels. (**c**) XRD pattern of hydrogels.

The chemical structure of the PVA hydrogel and PVA/ferritin biocomposite hydrogels was analyzed after freeze-drying. The FTIR spectra of the hydrogels are shown in Figure 2a. The band at 3278 cm^−1^ is attributed to the stretching peak of the hydroxyl group (-OH) of the PVA [44]. The absorption peak at 2941 cm^−1^ corresponds to the C-H stretching asymmetric. At 2906 cm^−1^, a stretching peak associated with the -CH-CH_2_ group was observed. In addition, the band at 1656 cm^−1^ corresponds to the acetyl C=O bond stretching oscillations [45]. It can be observed that as the concentration of ferritin increases from 1 mg/mL to 5 mg/mL, the absorption peak at 1656 cm^−1^ gradually strengthens. This can be attributed to the amide groups in proteins, which enhance the absorption of C=O bonds [46]. Furthermore, as shown in Figure 2b, the band at 1141 cm^−1^ is characteristic of the symmetric stretching vibration peak of the O-C-C bond, which is related to the crystallization of PVA [47]. The band at 1085 cm^−1^ is ascribed to the antisymmetric stretching vibration peak of the O-C-C bond belonging to the amorphous phase. It can be observed that as the concentration of ferritin increased to 5 mg/mL, the crystallization peak of PVA-Fer5 at 1141 cm^−1^ was significantly weakened, indicating that the crystallinity of PVA was greater than that of PVA-Fer5. This may be due to the hydrogen bonding interaction between ferritin and PVA, which reduces the crystallinity of PVA during the preparation process. The results indicate that as the concentration of ferritin increased, there were more hydrogen bonds between ferritin and PVA chains, resulting in a decrease in the crystallinity of PVA chains. Hydrogen bonding enables good interaction between ferritin and PVA chains, which is beneficial for improving the stretchability of the PVA/ferritin biocomposite hydrogel.

To further evaluate the crystallinity of PVA in the PVA/ferritin biocomposite hydrogel, the characteristics of the crystalline phase of the hydrogel are analyzed by XRD after freeze-drying. As shown in Figure 2c, the obvious diffraction peak is detected at 19.5°, which can be attributed to the (101) crystal plane of the semi-crystalline PVA hydrogel [48]. As the concentration of ferritin increases, the intensity of the diffraction peaks gradually decreases. It can be clearly observed that the intensity of the PVA-Fer5 hydrogel with a ferritin concentration of 5 mg/mL is lower than that of the PVA hydrogel. The results indicate that an increase in ferritin concentration could reduce the crystallinity of the PVA chains, which is consistent with the results of FTIR. The decrease in crystallinity may be related to the disordered distribution of ferritin between PVA chains. This further illustrates that the hydrogen bonding between PVA and ferritin reduces the transition of PVA from the amorphous to the crystalline phase. The crystallization of PVA could improve the stiffness but reduce the flexibility of the hydrogel. In this work, the flexibility of the PVA/ferritin biocomposite hydrogel will be improved by introducing ferritin into the PVA hydrogel.

### 2.3. Micromorphology of PVA/Ferritin Hydrogel

**Figure 3:**
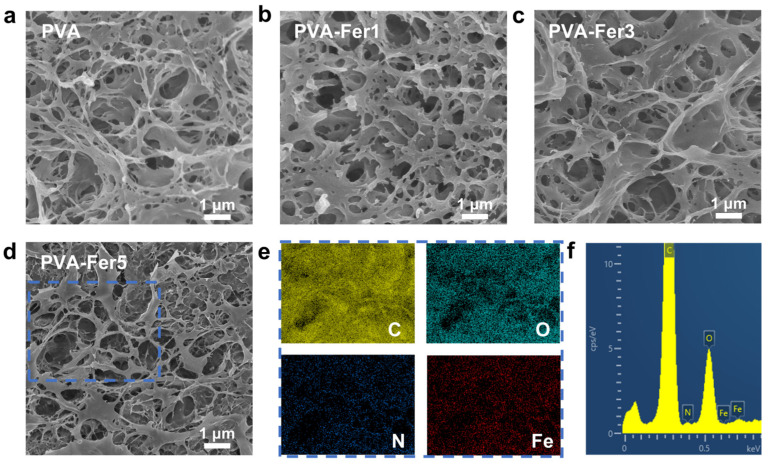
Microstructure of hydrogels. (**a**) PVA hydrogel. (**b**) PVA-Fer1 hydrogel. (**c**) PVA-Fer3 hydrogel. (**d**) PVA-Fer5 hydrogel. (**e**) Corresponding EDX element mappings of C, O, N, and Fe in PVA-Fer5 hydrogel. (**f**) Corresponding EDX spectrum.

The microstructure of hydrogels is detected by means of liquid nitrogen quenching and freeze-drying methods. During the freeze-drying process, the internal structure of the hydrogel may change slightly, but scanning electron microscope images can still be used to compare the structural changes between different hydrogel samples under the same freeze-drying process conditions. As shown in Figure 3a, the PVA hydrogel has a porous 3D network structure. As shown in Figure 3b–d, when the concentration of ferritin increases to 1, 3, and 5 mg/mL, respectively, the structure of the PVA/ferritin biocomposite hydrogel has no obvious changes and still maintains the porous 3D network structure. This is because PVA (6 wt.%), as the main component of the hydrogel, can form a crosslinked network structure after repeated freezing and thawing. A small amount of ferritin added to the hydrogel could be enveloped by the PVA network. This porous 3D network structure can deform under force thus endowing the PVA/ferritin biocomposite hydrogel with stretchability under rich water conditions. In addition, with the increase in ferritin concentration to 5 mg/mL, there are no agglomerates in the porous PVA network structure, indicating the productive complexation and compatibility of the ferritin and PVA chains. The element mappings of the PVA-Fer5 hydrogel are shown in Figure 3e. It can be observed that C, O, N, and Fe are evenly distributed in the network structure. Among them, N comes from amino acid of ferritin, while Fe ascribes to the inorganic iron core stored in ferritin. In addition, as shown in Figure 3f, the clear N and Fe elemental peaks can also be observed from the corresponding EDX spectrum. The element analysis of the hydrogel shows that N and Fe are uniformly distributed in the PVA network structure, indicating there was no obvious agglomeration of ferritin. The uniform dispersion of ferritin in the PVA network structure is conducive to the improvement of tensile properties of the PVA/ferritin biocomposite hydrogel. As a result, a PVA/ferritin biocomposite hydrogel with a porous 3D network structure and good uniformity is obtained.

### 2.4. Mechanical Properties of PVA/Ferritin Hydrogel

**Figure 4:**
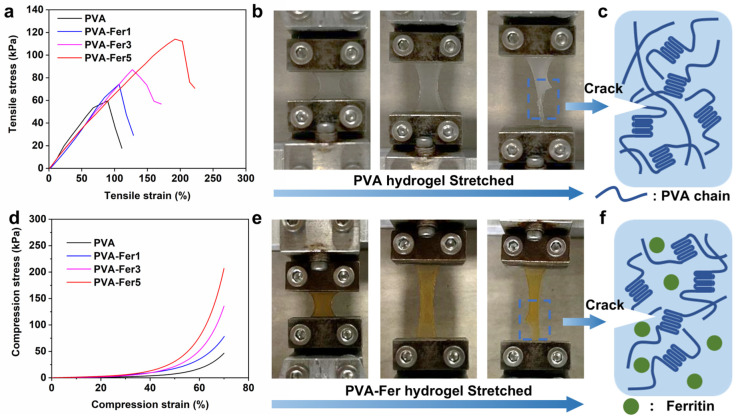
Mechanical properties of hydrogels. (**a**) Tensile stress–strain curve of hydrogels. (**b**) Photo of PVA hydrogel stretched. (**c**) Schematic diagram of PVA hydrogel fracture. (**d**) Compression stress–strain curve of hydrogels. (**e**) Photo of PVA-Fer5 hydrogel stretched. (**f**) Schematic diagram of PVA-Fer5 hydrogel fracture.

The 3D network structure allows the hydrogel to have a larger deformation space when subjected to external loads, leading the PVA/ferritin biocomposite hydrogel to be stretchable. In addition, hydrogels need to have good tensile properties to meet the strain sensor application [49,50]. In this study, the effect of ferritin on the tensile properties of PVA/ferritin biocomposite hydrogels is studied. As shown in Figure 4a, from the tensile stress–strain curve, it can be observed that with the increase in ferritin concentration, the tensile property of the hydrogel gradually increases. The fracture strain of the PVA hydrogel is 90%, and the fracture stress is 59.5 kPa. After adding ferritin with a concentration of 1 mg/mL, the fracture strain increases to 106% and the fracture stress increases to 74.3 kPa. With the further increase in ferritin concentration to 5 mg/mL, the tensile property of the PVA-Fer5 hydrogel is significantly increased, the fracture strain is 203%, and the fracture stress is 112.2 kPa. The tensile test results show that ferritin can effectively improve the tensile properties of PVA hydrogels. This is because the α-helix structure of a ferritin shell can withstand mechanical forces like a spring. During the tensile deformation of the hydrogel, the ferritin dispersed in the PVA network forms a hydrogen bond and acts as a spring model to bear the force, resulting in the improvement of the tensile property of the hydrogel. Compared to other methods, such as the use of graphene inorganic materials for toughening, the addition of a large number of inorganic materials can only improve the limited tensile properties of PVA-based hydrogels (fracture stress 65 kPa and fracture strain 180%) [16]. However, in this work, only a small amount of ferritin can significantly improve the tensile properties of PVA-based hydrogels. This is due to the productive complexation, compatibility, and adaptability of ferritin in PVA, which makes ferritin have a significant toughening effect.

In addition, the fracture toughness of the PVA hydrogel and PVA/ferritin biocomposite hydrogels is calculated by integrating the stress–strain curve. As shown in Table 1, the fracture strain, fracture stress, and fracture toughness of the different hydrogels are listed. It can be calculated that the toughness of the PVA hydrogel is 39.17 kJ/m^3^. With the increase in ferritin concentration, the fracture toughness of the PVA/ferritin biocomposite hydrogel increased gradually. When the concentration of ferritin is 5 mg/mL, the fracture toughness of the PVA-Fer5 hydrogel is significantly increased to 147.03 kJ/m^3^, more than 3 times that of PVA hydrogel (39.17 kJ/m^3^). Therefore, it can be concluded that ferritin as a toughening component of nano cage can effectively improve the fracture toughness of the PVA hydrogel. Ferritin is a cage-shaped protein composed of 24 α-helix subunits with a diameter of approximately 12 nm. The hydrogen bond interaction between ferritin and PVA chains and the unique nano cage structure of ferritin improves the tensile properties of the PVA/ferritin biocomposite hydrogel. The tensile property of the PVA/ferritin biocomposite hydrogel can be enhanced through this single effective toughening component and facile freezing–thawing method. The results indicate that the PVA/ferritin biocomposite hydrogel has good stretchability, toughness, and flexibility.

Changes in the PVA hydrogel and PVA-Fer5 hydrogel during stretching are observed. As shown in Figure 4b, the crack propagation during the tensile fracture can be observed from the photos of the PVA hydrogel. The schematic diagram shows the crack propagation caused by the fracture of PVA chains (Figure 4c). As shown in Figure 4e, during the tensile process of the PVA/ferritin biocomposite hydrogel, the crack expansion is effectively slowed down, which improves the tensile properties of the hydrogel. Ferritin is evenly dispersed in the hydrogel and interacts with PVA chains to form hydrogen bonds, improving the toughness of the hydrogel. The effect of ferritin on the mechanical properties of hydrogels was further studied by a compression test. As shown in Figure 4d, when the compression strain reaches 70%, the compression stress of the PVA hydrogel is 46.5 kPa. The compressive stress of PVA-Fer5 increased to 206.9 kPa. The results also show that ferritin can effectively improve the mechanical properties of hydrogels. As a result, the good tensile property enables the PVA/ferritin biocomposite hydrogel to bear the deformation caused by joint movement when applied to human skin.

### 2.5. Performances and Applications of PVA/Ferritin Hydrogel

**Figure 5:**
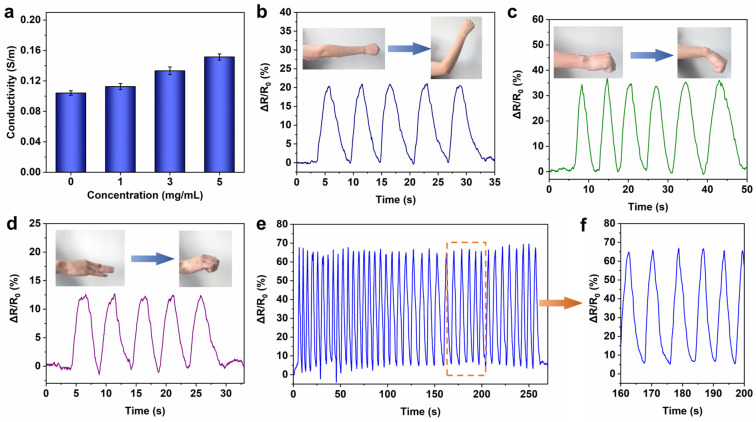
Strain sensing performance of hydrogels. (**a**) Conductivity of hydrogels. (**b**) Elbow movement monitoring. (**c**) Wrist movement monitoring. (**d**) Finger movement monitoring. (**e**) Cyclic working stability of PVA/ferritin hydrogel strain sensor. (**f**) Resistance response curve during cyclical working.

When used as a strain–resistance response in flexible sensors, hydrogels need to have good conductivity [51,52]. In this work, the conductivity of ferritin-enhanced PVA/ferritin biocomposite hydrogels is tested. As shown in Figure 5a, the conductivity of the PVA hydrogel is 0.1 S/m. When the concentration of ferritin is 5 mg/mL, the conductivity of the PVA-Fer5 hydrogel increases to 0.15 S/m. The results indicate that ferritin can improve the conductivity of hydrogels. This is because the inorganic iron core in ferritin contains iron ions, and the protein provides ion free residue, leading to the improvement of ionic conductivity of hydrogels [53]. In this work, the natural ferritin with a unique nano cage structure and inorganic iron core composition is used to effectively improve the tensile property and ionic conductivity of the PVA/ferritin biocomposite hydrogel. This effective strategy is beneficial for flexible electronic sensing applications.

**Figure 6:**
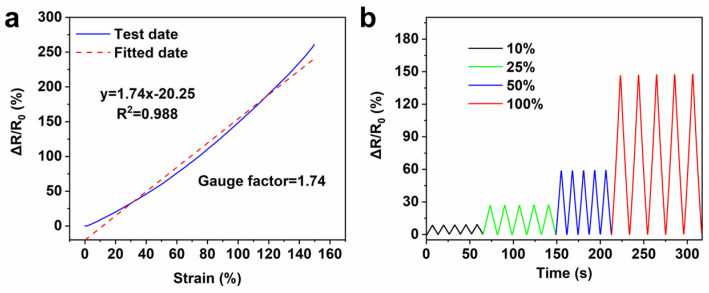
Sensitivity and step response–recovery performances of hydrogels. (**a**) Linearity of relative resistance as function of strain. (**b**) Relative resistance curve under different strains.

Compared to other samples, the PVA-Fer5 hydrogel has the highest fracture strain of 203%, the highest stress of 112.2 kPa, and the highest conductivity of 0.15 S/m. Based on the above analysis, it can be concluded that the tensile strength and ionic conductivity of the hydrogel with 5 mg/mL ferritin is the best, which could be applied in the field of flexible strain sensors for monitoring the movement of various parts of the human body. In order to study the application of hydrogel in human activity monitoring, the two ends of the PVA-Fer5 hydrogel are, respectively, connected with copper wire and then packaged with VHB for strain sensor applications. This encapsulation can prevent the moisture of the hydrogel from volatilizing in use and provide a better fit for the skin surface. The sensitivity of the hydrogel strain sensor was measured. The gauge factor is calculated from the linearity of the relative resistance as a function of strain. As shown in Figure 6a, the fitting correlation coefficient R^2^ is as high as 0.988. When the strain is 150%, the gauge factor value reaches 1.74. The results show that the PVA/ferritin hydrogel strain sensor has good sensitivity. The stability of the hydrogel sensors was assessed by stretching under different levels of strain. As shown in Figure 6b, the resistance response can be stably and accurately detected during repeated stretching in different strain ranges (10%, 25%, 50%, and 100%), indicating the stable step response–recovery performances of the PVA/ferritin hydrogel sensor. In addition, as shown in Figure 5b, when the PVA/ferritin hydrogel strain sensor is attached to the elbow, the hydrogel can generate a resistance signal response with the swing of the elbow. When the elbow is bent upward, the hydrogel is stretched, and the relative resistance of the hydrogel becomes larger and responds in real-time. In addition, as shown in Figure 5c,d, when applied to the wrist or finger, the PVA/ferritin hydrogel strain sensor also shows a stable electrical signal response in real-time. The results indicate that the PVA/ferritin biocomposite hydrogel as strain sensor can be used to accurately monitor the movement of different joints in real-time. Furthermore, the stability of PVA/ferritin hydrogels in long-term strain–resistance response is further evaluated. As shown in Figure 5e, the PVA/ferritin hydrogel strain sensor exhibits stable resistance changes during multiple cycles within 4 min. As shown in Figure 5f, it can be observed that the relative resistance change curve remains stable, indicating the anti-fatigue performance of the PVA/ferritin biocomposite hydrogel. As shown in Table 2, compared to the conductivity, working strain, and gauge factor of other PVA-based hydrogel strain sensors, the PVA/ferritin biocomposite hydrogel shows good sensing performance. The results indicate that when used as flexible electronic materials for human joint movement monitoring, PVA/ferritin biocomposite hydrogel shows accuracy, real-time sensing, and stability, which is expected to be applied in the field of wearable electronics.

## 3. Conclusions

In summary, a PVA/ferritin biocomposite hydrogel was prepared by a repetitive freeze–thaw method. The biocomposite hydrogel has a three-dimensional network structure and uniform dispersion of ferritin. Ferritin can interact with PVA chains through hydrogen bonding, reducing the crystallinity of the PVA. As a result, the tensile test indicated that the stretchability of the hydrogel is enhanced. With the increase in ferritin concentration to 5 mg/mL, the fracture strain of the PVA/ferritin hydrogel increased from 90% to 203%, and the fracture stress increased from 59.5 kPa to 112.2 kPa. The fracture toughness of the PVA/ferritin hydrogel was significantly enhanced to 147.03 kJ/m^3^, more than 3 times that of the PVA hydrogel (39.17 kJ/m^3^). In addition, the PVA/ferritin biocomposite hydrogel exhibited enhanced conductivity (0.15 S/m) and good sensitivity (gauge factor = 1.7 at 150% strain). A hydrogel with such properties is an ideal candidate for strain sensors. The PVA/ferritin hydrogel as strain sensor showed real-time resistance response for joint activity monitoring. After long-term cyclic working, the PVA/ferritin hydrogel strain sensor exhibited good stability, as observed. This biocomposite hydrogel could provide great potential for application in wearable flexible electronic devices.

## 4. Materials and Methods

### 4.1. Materials

Polyvinyl alcohol (Mw~205,000, PVA) was purchased from Sigma-Aldrich (St. Louis, MO, USA). Ferritin from a horse spleen was purchased from Shanghai yuanye Bio-Technology Co., Ltd., (Shanghai, China). The used ultrapure water was prepared by the Millipore system (St. Louis, MO, USA).

### 4.2. Preparation of PVA/Ferritin Hydrogels

First, 10 g of PVA powder was added to 90 g of deionized water, and then the solution was stirred at 95 °C for 2 h. After cooling to room temperature, 10 wt% PVA solution was obtained. The ferritin solution and ultrapure water were added to 5 g of the PVA solution with continuous stirring at room temperature. The mass of the final PVA in the solution was diluted to 6 wt%. The final concentration of ferritin added to the solution is 1, 3, and 5 mg/mL, respectively. The PVA/ferritin mixture was poured into a polytetrafluoroethylene (PTFE) mold with a length of 30 mm, width of 6 mm, and depth of 2 mm. The PTFE mold was degassed by sonication for 30 min to remove bubbles from the solution. The PTFE mold was frozen at −20 °C for 12 h and then thawed at room temperature for 12 h. The PVA/ferritin hydrogels were obtained after repeated freezing and thawing 3 times. The hydrogels with 1, 3, and 5 mg/mL ferritin were named PVA-Fer1, PVA-Fer3, and PVA-Fer5, respectively.

### 4.3. Characterization of Hydrogels

The hydrogel was quenched by liquid nitrogen for 5 min and then freeze-dried in a freeze dryer (LGJ-12, Beijing Songyuan Huaxing Technology Development Co., Ltd., Beijing, China) for 2 days. Then, the sample was sprayed with platinum and the structure of the sample was observed by a scanning electron microscope (SEM, SU8230, Hitachi, Ltd., Tokyo, Japan) with an accelerating voltage of 5 kV. The FTIR absorption spectra of the samples were measured by Fourier transform infrared spectroscopy (FTIR, Thermo Nicolet Nexus, Ontario, ON, Canada) with a wavelength range of 4000–400 cm^−1^. The phase of the samples was characterized by X-ray diffraction (XRD, PANalytical-Empyrean X-ray diffractometer equipped, Malvern Panalytical, Malvern, UK) from 10° to 80°.

### 4.4. Mechanical Property Test of Hydrogels

In the tensile test, the hydrogel size is 30 mm in length, 6 mm in width, and 2 mm in depth. The hydrogel was stretched by a PI servo testing machine with a 5-N load cell at a speed of 0.2 mm/s and ambient temperature. The fracture toughness of hydrogels is calculated by integrating the stress–strain curve. In the compression test, the hydrogel size is 14 mm in diameter and 7 mm in height. The hydrogel is compressed by a device (Instron-5967) with a 50-N load cell at a speed of 2 mm/min and ambient temperature.

### 4.5. Conductivity Test of Hydrogels

The resistance (R) of the hydrogel was measured by an LCR meter (TH2830, Changzhou Tonghui Electronic Co. Ltd., Changzhou, China). Then, the conductivity (σ) was defined as follows:

σ = L/RS

where R is the resistance of the hydrogel, S is the length between two electrodes, and L represents the cross-sectional area of contact between the hydrogel and electrode.

### 4.6. Strain Sensing Performance Test of Hydrogels

The PVA-Fer5 hydrogels with 5 mg/mL ferritin were used as strain sensors. The two ends of the PVA/ferritin hydrogel with a length of 30 mm, width of 6 mm, and depth of 2 mm were, respectively, connected with copper wire and packaged with VHB as strain sensors. Then, the copper electrodes at both ends were connected to an LCR meter (TH2830, Changzhou Tonghui Electronic Co. Ltd., Changzhou, China) controlled by a LabView program for collecting real-time resistance signals. The hydrogel strain sensor was attached to the joint for monitoring activity. The relative resistance change was defined as follows:

ΔR/R_0_ = (R − R_0_)/R_0_ × 100%

where R_0_ is the original resistance and R is the resistance in real-time.

The gauge factor (GF) was defined as follows:

GF = (ΔR/R_0_)/ΔL

where ΔL is the strain of the strain sensor. It is calculated by the slope of the relative resistance–strain response curve.

For a long-term working test, the hydrogel strain sensor is attached to the wrist, and the stability of the hydrogel is tested by repeatedly bending the wrist.

## Figures and Tables

**Table 1:** The comprehensive mechanical properties of the hydrogels.

Samples	Fracture Strain (%)	Fracture Stress (kPa)	Fracture Toughness (kJ/m^3^)
PVA	90	59.5	39.17
PVA-Fer1	106	74.3	50.8
PVA-Fer3	127	87.2	87.65
PVA-Fer5	203	112.2	147.03

**Table 2:** Comparison of conductivity, working strain, and gauge factor of this work with other PVA-based hydrogel strain sensors.

Hydrogel	Conductivity (S/m)	Working Strain (%)	Gauge Factor	Ref.
PVA-CNF-ZnSO_4_	0.32	200	1.7	[54]
PVA-PAA-CNT-PEDOT:PSS-Fe^3+^	-	100	1.16	[55]
PVA-PVP-CNCs-Fe^3+^	-	200	0.478	[56]
PVA/PSBMA	0.037	300	1.5	[57]
PVA/GO	3.38	100	2.05	[16]
PVA/Ferritin	0.15	150	1.7	This work

## Data Availability

The original contributions presented in this study are included in the article. Further inquiries can be directed to the corresponding author.
